# Mortality surveillance as an early warning system for respiratory infection outbreaks: lessons from the COVID-19 pandemic in Alexandria, Egypt

**DOI:** 10.1186/s42506-025-00205-y

**Published:** 2026-02-23

**Authors:** Rehab Meckawy, Heba M. T. El Weshahi, Eman Foda, Eman A. Sultan

**Affiliations:** Community Medicine Department, Faculty of Medicine, Alexandria University, Alexandria, Egypt

**Keywords:** Mortality surveillance, Early warning system, COVID-19, Respiratory failure, LMICs

## Abstract

**Background:**

The COVID-19 pandemic highlighted critical gaps in mortality surveillance, particularly in low- and middle-income countries (LMICs). This study evaluated the feasibility of using routine mortality data as an early warning system for respiratory outbreaks in Alexandria, Egypt.

**Methods:**

A retrospective time-series analysis of 61,378 deaths (2017–2022) was conducted. Respiratory failure (ICD-10: J96.90) was used as a proxy for COVID-19-related mortality. As population denominators were unavailable at subdistrict level, analyses relied on proportionate mortality, reflecting the pragmatic data scope of the current registry system. Expected deaths were estimated using an exponential smoothing model, and excess mortality was identified when observed deaths exceeded expectations for three or more consecutive months.

**Results:**

Deaths attributed to respiratory failure increased notably during 2020–2021, with sustained excess mortality signals corresponding to pandemic peaks. Mortality among adults aged 70–75 years rose markedly, and total years of life lost during the pandemic reached 41,819 across high-risk age groups (40–70 years).

**Conclusions:**

Routine mortality data can serve as a practical foundation for early detection of respiratory disease outbreaks in resource-limited settings. Future investments should focus on strengthening human resources, expanding digital infrastructure, and improving data standardization to ensure the long-term sustainability and scalability of mortality surveillance systems in LMICs.

**Supplementary Information:**

The online version contains supplementary material available at 10.1186/s42506-025-00205-y.

## Introduction

The COVID-19 pandemic represented one of the most significant global health emergencies in recent history. Its rapid transmission and profound health impacts underscore the urgency of developing effective prevention and response strategies for emerging diseases globally [1]. The World Health Organization (WHO) declared COVID-19 a pandemic on March 11, 2020, marking its global recognition [2].

Estimates by *The Economist* suggest that actual COVID-19–related deaths could be two to four times higher than reported figures, potentially reaching 20–25 million globally [3]. These excess deaths result not only from the virus’s direct effects but also from indirect consequences, such as overwhelmed healthcare systems in both high- and low-resource settings [4]. These indirect impacts on other mortality causes are termed Other Pandemic-Related Mortality (OPRM) [5].

Egypt reported its first COVID-19 case on February 14, 2020, and imposed containment measures by March 17, including closure of educational institutions and public venues [6]. By May 2023, Egypt’s official COVID-19 death toll had reached approximately 25,000 [7]. The pandemic heavily disrupted the Egyptian economy, notably halting tourism, which contributes around 12% of GDP and 10% of employment [8].

Surveillance—defined as the continuous collection, analysis, and dissemination of health data—is vital for public health planning and timely intervention [9]. However, in Egypt and other LMICs, the lack of timely, accurate surveillance data hindered early recognition of COVID-19–related deaths [10].

Early Warning Systems (EWSs) use health informatics data from sources like emergency departments, public health records, pharmacies, and death registries to detect infectious disease outbreaks [11, 12]. However, LMICs often lack the infrastructure to support robust, real-time surveillance systems, relying primarily on diagnosis-based or mortality data rather than comprehensive syndromic surveillance [12, 13]. Standard outbreak detection methods such as Serfling-type regression [14], Farrington/Noufaily algorithms [15], EuroMOMO z-scores [16], and EARS C1–C3 [17] have been widely used in high-income countries. While these provide robust excess-mortality estimation, their complexity and data requirements pose challenges in LMICs.

Detecting unexpected mortality spikes—before diagnostic confirmation—can offer crucial early outbreak signals [18]. In this context, mortality surveillance can also serve an early warning function, particularly where diagnostic or syndromic surveillance data are delayed or incomplete. As noted by Rao et al., “*Mortality surveillance is essential for early warnings and the monitoring of the incidence and progress of epidemics*,* as well as guiding the public health response*” [19]. Within LMIC settings, mortality-based signals can therefore provide secondary early indicators of outbreak escalation when other data streams are limited.

This retrospective observational time-series study is the first to assess the practicality of mortality surveillance for outbreak detection in Alexandria, Egypt. Focusing on deaths from respiratory failure—a primary cause of COVID-19 mortality—we evaluated deviations from expected trends and analyzed demographic patterns (age, sex, marital status) in mortality data during the pre- and intrapandemic periods. The study also calculated the burden of premature COVID-19–related mortality using the years of life lost (YLL) metric. This study focuses on mortality patterns associated with respiratory failure, reflecting outbreaks driven primarily by respiratory infections such as COVID-19, where respiratory manifestations dominate clinical outcomes.

## Methods

### Data sources for surveillance

Mortality data were extracted from structured death registry transfer sheets provided by the Egyptian Ministry of Health and Population (MoHP; see additional file 1). These sheets are derived from official death certificates, which are coded according to ICD-10 guidelines and digitally entered into the national civil registration system [20, 21]. The direct cause of death, including respiratory failure, was recorded based on hospital discharge reports, which served as the clinical basis for certification. This adds a degree of diagnostic reliability to the coding process. However, we acknowledge that respiratory failure (ICD-10 code J96.90) [22] remains a non-specific endpoint, and may not capture the full scope of COVID-19–related mortality.

This study included all routinely recorded deaths from a pragmatically selected subset of eight health offices in Alexandria between 2017 and 2022. Selection was guided by registry accessibility, completeness of cause-of-death data, and geographic representation across Alexandria’s administrative districts. Specific office names were anonymized by the Ministry of Health and Population; however, district-level distribution is provided in Table 1 to illustrate contextual coverage. Demographic variables—age, gender, and marital status—were collected alongside the recorded cause of death. Respiratory failure (ICD-10: J96.90) was used as a pragmatic proxy for COVID-19–related mortality in our analysis due to the phased and the relatively delayed national implementation of specific COVID-19 codes (U07.1 and U07.2) during 2020. Michael Willie’s 2021 study [23] exemplifies the period during which these codes were adopted for classifying COVID-19 diagnoses, underscoring that their conceptual relevance remains but that retrospective analyses may be affected by their staggered introduction. ^‘^

**Table 1:** Number of health offices per district in Alexandria, Egypt (reported by key informants)

District	Number of health offices
El Agamy El Gomrok El Montaza West Middle East Al Amreya Borg El Arab	4 4 4 6 9 11 13 18
Total	69

The eight offices included in this study were pragmatically selected from within these districts based on registry accessibility and completeness. Specific office identifiers were anonymized for confidentiality under Ministry of Health data access protocols

Although broader respiratory categories—such as J09–J18 (influenza and pneumonia) and J80 (acute respiratory distress syndrome)—were present in a subset of records, these codes were inconsistently applied across health offices and years, precluding their inclusion in the main time-series analysis. A feasibility review of 2021 data showed that these additional respiratory causes represented fewer than 5% of all recorded deaths. This supports the validity of J96.90 as a practical and stable proxy indicator for mortality surveillance in this setting.

The implications of potential misclassification are further addressed in the discussion. Data from 2022 were shown descriptively to contextualize postpandemic trends, but were not part of forecasting analyses.

### System coverage

The MoHP centrally manages Egypt’s death registry, which mandates notification of all deaths under national law. The system integrates demographic and cause of death information and uses ICD-based classification standards to maintain consistent nationwide records [20]. Death registrations are legally mandated within 48 h. Records were cross-checked with national ID numbers to prevent duplication. While this provides a comprehensive mortality database, accuracy may be constrained by clinical and procedural variability in cause of death certification, particularly under conditions of limited diagnostic capacity.

### Study population and statistical analysis

We described the distribution of deaths from 2017 to 2021 (pre- and intrapandemic periods) by age, gender, and marital status. Descriptive and inferential statistical methods were used to analyze these trends. Between-group comparisons for proportionate mortality were expressed as Risk Differences and Rate Ratios with 95% confidence intervals. Population denominators were not accessible under the current ethical approval and registry framework. Therefore, the study relied on proportionate mortality as a feasible and standardized indicator of relative mortality burden within available routine data, acknowledging that this approach cannot capture absolute rate changes. These effect sizes provide more clinically meaningful measures of association than statistical significance testing for the comparisons of interest. Years of life lost (YLL) were calculated using WHO/GBD remaining life expectancies at age group midpoints for high-risk groups (40–70 years), providing more accurate estimates of premature mortality burden [24].

### Detection of excess mortality and system performance

A pilot study was conducted to test feasibility, tool usability, and fieldwork logistics. The pilot highlighted a critical capacity gap: most surveillance personnel had limited training in advanced statistical methods. Accordingly, we adopted a simplified analytic strategy focusing on accessible numerical comparisons between observed and expected deaths, facilitating local uptake. Simplicity and acceptability—defined by the CDC as ease of use and willingness of personnel to participate—were prioritized to enhance reporting compliance and reduce data entry errors [25, 26]. This analysis is exploratory and designed to test the feasibility of using existing mortality data in LMIC contexts where real-time population denominators are often incomplete or unavailable.

To detect excess mortality, time-series forecasting was applied to model expected monthly deaths from October 2019 to December 2021, using ETS exponential smoothing. Expected monthly deaths were estimated using a seasonal time-series accounting for recurrent seasonal mortality patterns. Historical data from January 2017 to September 2019 served as the baseline to account for seasonal and temporal mortality patterns. Both all-cause and respiratory failure-specific mortality trends were analyzed. Forecast model adequacy was evaluated using the Ljung-Box Q test, with a non-significant p-value (*P* > 0.05) indicating randomness of residuals and acceptable model fit.

### Usefulness and contextual relevance

Although respiratory failure was our primary endpoint, all-cause mortality was also assessed to account for Other Pandemic-Related Mortality (OPRM), which includes deaths indirectly attributable to COVID-19 [5]. The surveillance system was considered to flag a potential signal if excess respiratory failure mortality persisted for greater than or equal to three (≥ 3) consecutive months. This threshold was not intended as a confirmatory outbreak definition, but rather as a screening indicator to identify sustained deviations from expected mortality trends using methods that are easily interpretable by local surveillance staff. This threshold balances epidemiological rigor with operational feasibility in LMICs surveillance, accounting for short-term fluctuations and aligning with WHO surveillance guidance [27]. The WHO, CDC, and UNHCR guidelines were used to frame our analytic strategy, all of which emphasize data timeliness, usability, and the value of basic statistical tools when infrastructure and workforce capacity are constrained [28–30].

### STROBE and RECORD compliance declaration

This study complies with the STROBE (Strengthening the Reporting of Observational Studies in Epidemiology) [31] guidelines for observational research, specifically for a retrospective time-series design. In addition, it follows the RECORD (The REporting of studies Conducted using Observational Routinely-collected health Data) [32] extension due to the use of routinely collected mortality registry data from the Ministry of Health and Population in Egypt. Data sources and completeness were described, and data cleaning procedures, including duplicate resolution and handling of missing data, were applied. The study acknowledges limitations related to representativeness, given that the data were obtained from eight registry offices.

## Results

### Overview of mortality trends

A total of 61,378 deaths were recorded from 2017 to 2022 across eight pragmatically selected health offices in Alexandria, each representing a distinct health district. Selection was pragmatic, driven by registry access rather than full randomization; therefore, the findings reflect surveillance feasibility rather than population-representative estimates. Among these, 9,497 deaths (15.47%) were attributed to respiratory failure (ICD-10 code J96.90), as recorded on death certificates based on hospital discharge diagnoses.

The proportionate mortality from respiratory failure increased substantially from 13.69% in 2019 to 25.64% in 2021, representing an absolute increase of 11.95% points (Risk Difference = 0.119, 95% CI: 0.113 to 0.126) and nearly doubling the mortality rate (Rate Ratio = 1.87, 95% CI: 1.75 to 2.00). Rates in 2022 differed significantly from 2020 to 2021, but not from 2018 to 2019, suggesting stabilization in the postpandemic period.

### Data source and quality assessment

In accordance with RECORD guidelines, we conducted a systematic quality assessment. The initial extraction yielded 61,808 raw records. After resolving duplicates based on a composite key (ID, date of death, health office), which affected 0.7% (*n* = 430) of records, the final analytical cohort contained 61,378 unique deaths. The completeness of core variables was high: Date of Death (99.8%), Cause of Death (98.5%), and Health Office (99.9%).

### Demographic distribution of mortality

The mean age at death increased over the years: from 60.5 years in 2017 to 63.9 years in 2021. The majority of deaths occurred among those aged 70 years and above, particularly in 2020 (1,693 deaths) and 2021 (1,819 deaths). Deaths among adults aged 70–75 years increased from 13.4% in the pre-pandemic period to 15.3% during the pandemic, representing a 1.87% point increase (Risk Difference = 0.019, 95% CI: 0.014 to 0.023).

Sex-based comparisons revealed no significant differences in respiratory failure mortality between males and females during the pre- and intrapandemic periods (*P* = 0.308), despite a slight male predominance in 2017 (58.83%) and 2020 (58.94%). A higher proportion of deaths occurred among individuals recorded as divorced during the intrapandemic period. This reflects a descriptive observation, without inference of increased risk.

### Time-series analysis – all-cause mortality

**Fig. 1:**
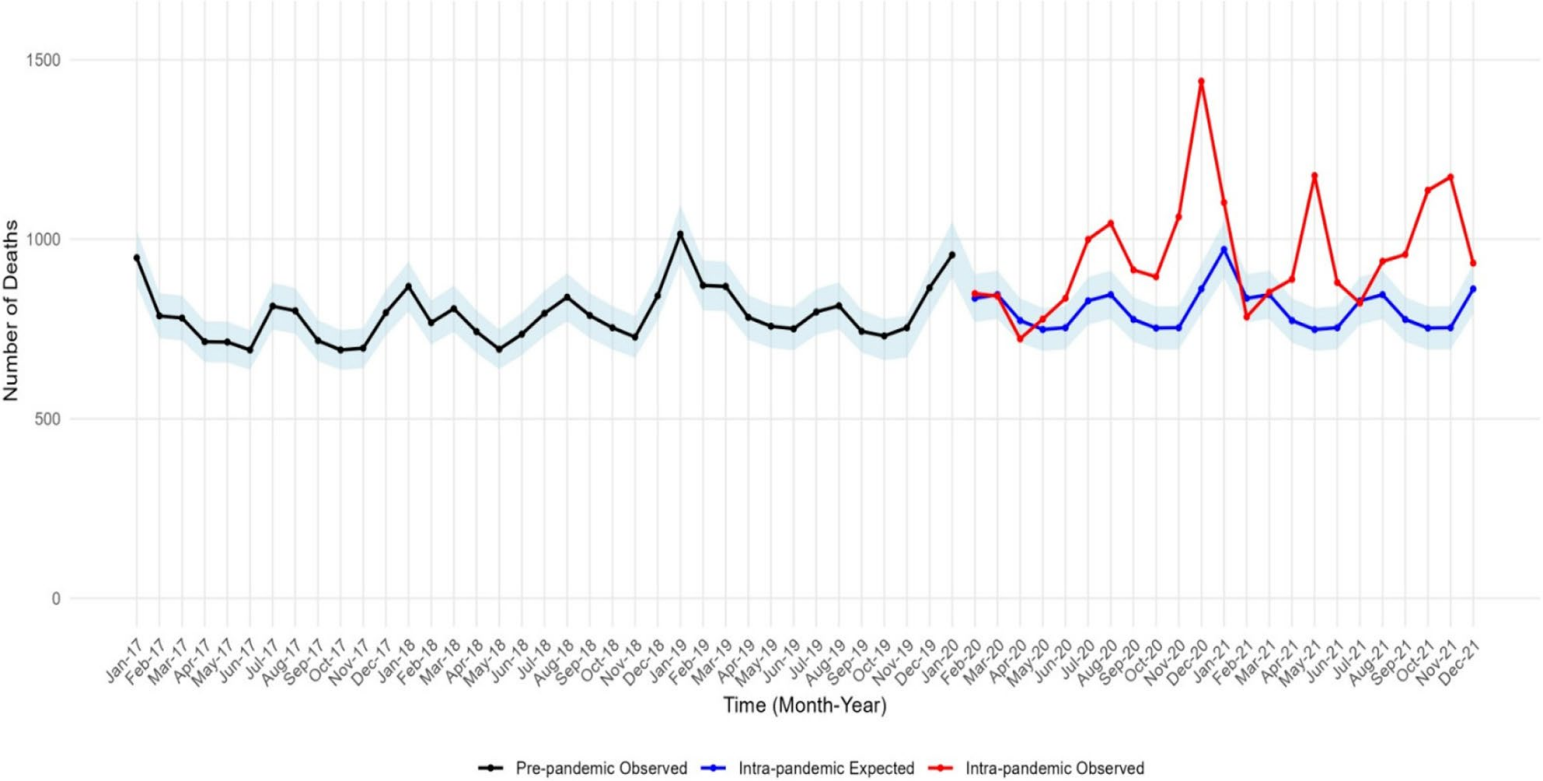
Monthly observed and expected total deaths in the studied health offices in Alexandria (2017–2021). The shaded area depicts the 95% prediction intervals. They are derived from the ETS model and serve as a preliminary illustration

The time-series analysis of all-cause mortality from 2017 to 2021 revealed distinct patterns before and during the COVID-19 pandemic, as illustrated in Fig. 1. Beginning in March 2020, a clear divergence emerged between the observed deaths and the expected baseline. Several pronounced peaks in observed mortality substantially exceeded the model’s forecast.

Critically, the inclusion of the 95% prediction interval provides a statistical benchmark for identifying periods of significant excess mortality. The interval represents the range within which future observations were expected to fall, based solely on the historical pattern and its inherent variability. Observed mortality consistently breached the upper bound of the 95% prediction interval during specific months. Most notably, a major peak occurred in December 2020, where observed deaths far surpassed the expected range. Other periods, such as the early months of 2021 (e.g., January, May), also showed observed values positioned well above the prediction band.

The fact that these peaks lie outside the prediction interval indicates that the observed mortality was not merely a high random fluctuation but represented a statistically significant excess. The vertical distance between the observed values and the upper prediction limit during these peaks quantifies the magnitude of this excess, which was substantial in several instances. The timing of these significant excess events aligns with known waves of COVID-19 in the region, suggesting a strong temporal association between the pandemic and the observed surge in all-cause mortality.

### Time-series analysis – respiratory failure mortality

Analysis of respiratory failure deaths showed the proportion occurring in October-December increased from 2.22% in 2017 to 2.74% in 2019 (Risk Difference = 0.005, 95% CI: 0.001 to 0.009), and from 3.08% to 3.90% in January-March between 2018 and 2020 (Risk Difference = 0.008, 95% CI: 0.004 to 0.012).

**Fig. 2:**
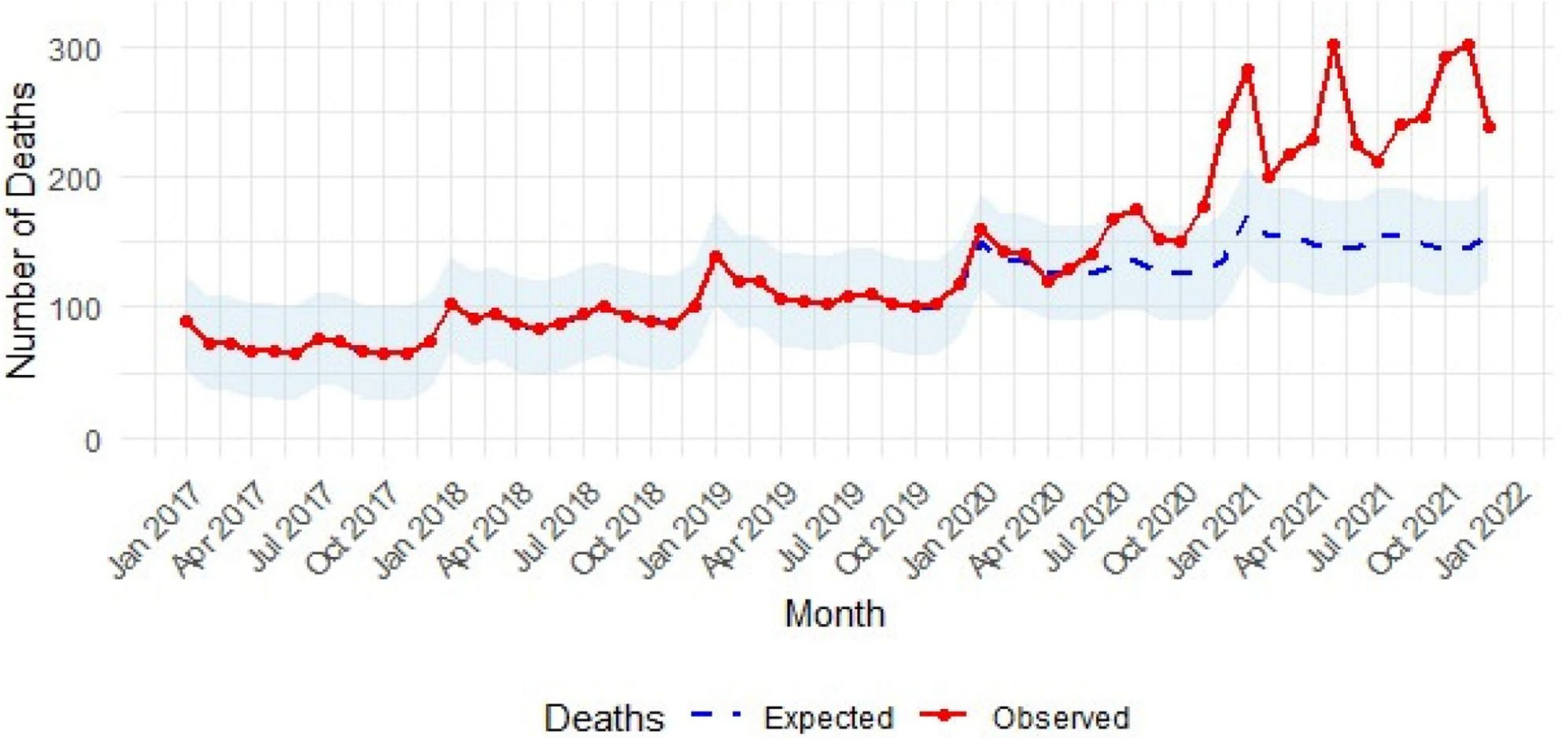
Monthly observed and expected respiratory failure deaths in the studied health offices in Alexandria (2017–2021). The shaded area depicts the 95% prediction intervals. They are derived from the ETS model and serve as a preliminary illustration

Forecasting based on prepandemic data (January 2017-September 2019) was applied to predict respiratory failure deaths through December 2021. The Ljung–Box Q test (*P* = 0.002) indicated residual autocorrelation and a poor model fit. Throughout the pandemic period, observed respiratory failure deaths consistently exceeded the upper bound of the 95% prediction intervals. This pattern indicates that the observed mortality increases were statistically significant and unlikely to represent random variation from pre-pandemic trends. The sustained breach of prediction interval boundaries, particularly from mid-2020 through 2021, provides strong evidence that the excess mortality represented a fundamental shift in respiratory disease epidemiology rather than expected statistical fluctuation (Figure 2).

A modest rise in observed deaths was noted from late 2019, likely reflecting seasonal respiratory mortality or under-recognition of early COVID-19–related deaths before formal case identification and coding began. The first sustained and statistically significant divergence from expected trends occurred from January 2020 onward, coinciding with the onset of the pandemic.

### Burden of COVID-19–related premature mortality

**Fig. 3:**
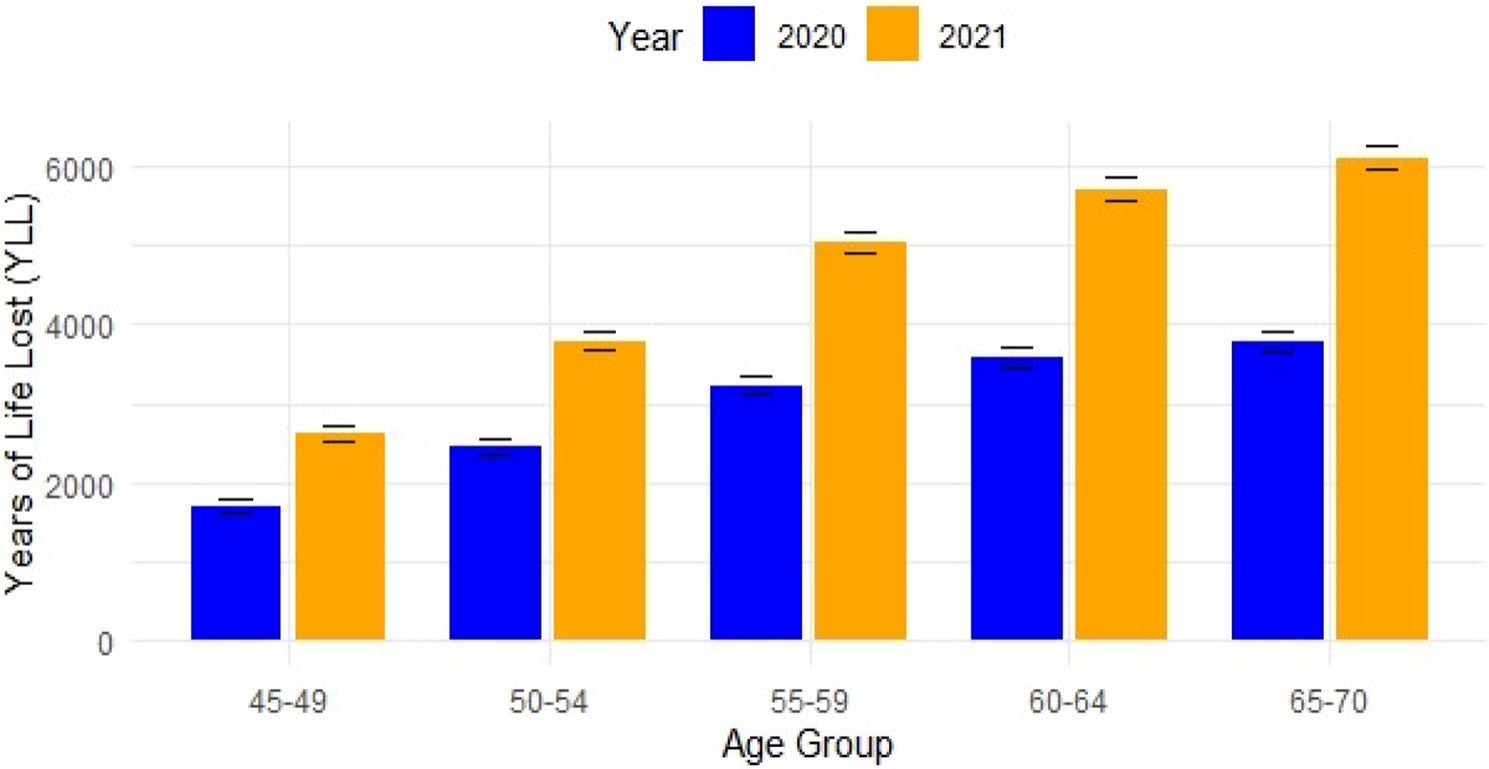
Years of life lost from respiratory failure among high-risk age groups. Error bars represent 95% uncertainty intervals calculated using Poisson distribution assumptions (SE = √YLL). Years of Life Lost (YLL) were estimated using WHO/GBD remaining life expectancies at age group midpoints

Intrapandemic Years of Life Lost (YLL) due to respiratory failure totaled 41,819 years across 2020 and 2021 among high-risk age groups (40–70 years). Annual totals rose from 16,181 YLL in 2020 to 25,638 YLL in 2021, reflecting a 58% increase in burden. The age group 65–70 consistently showed the highest YLL: 3,780 years in 2020 and 6,104 in 2021, with substantial burdens also observed in the 60–64 age group (3,587 YLL in 2020; 5,712 YLL in 2021) (Figure 3).

This concentration among middle-aged adults underscores the substantial societal and economic impact of respiratory failure–related COVID-19 mortality during the pandemic period.

## Discussion

This retrospective observational time-series study assessed the feasibility and performance of a mortality surveillance system for outbreak detection in Alexandria, Egypt. Using routinely collected death registry data, we identified significant deviations from expected mortality patterns, particularly in deaths attributed to respiratory failure (ICD-10 code J96.90), the dominant clinical endpoint of COVID-19. Time-series analysis revealed that sustained increases in respiratory failure deaths—lasting at least three consecutive months—offered an accessible and reliable early warning signal.

This approach, centered on simplicity and acceptability, aligns well with the analytic capacity of local health surveillance teams in LMICs. While the analysis used proportionate mortality due to the unavailability of disaggregated population data, this design reflects the operational reality of LMIC mortality surveillance, providing a realistic model for local health authorities to build from as data infrastructure improves.

Our findings show a sharp increase in respiratory failure–related mortality during 2020 and 2021, peaking at 25.64% of all deaths in 2021. This trend aligns with global data from the Global Burden of Disease study, which noted increased lower respiratory tract infection (LRI) mortality during the pandemic [33]. Additionally, monthly time-series analysis identified mortality spikes consistent with COVID-19 waves, demonstrating the surveillance system’s utility even in the absence of diagnostic confirmation. These mortality patterns are suggestive of pandemic-related impact, but cannot confirm causality without external validation.

The early exceedance signal observed in late 2019 should be interpreted with caution. This analysis was not designed to evaluate the performance of Egypt’s existing mortality surveillance system but rather to assess the feasibility of using routine registry data as an early warning tool. Given delays in diagnostic testing and the phased introduction of COVID-19–specific codes (U07.1/U07.2), some early COVID-19 deaths may have been recorded under nonspecific respiratory causes such as J96.90. This likely contributed to apparent pre-pandemic excesses in respiratory mortality.

### Demographic trends

Older adults, particularly those aged 70–75 years, echoing CDC reports of increased age-specific mortality in those 65 and older [34]. Although respiratory failure mortality was higher among males throughout the study period, sex-based differences during the pandemic years were not statistically significant. Similar findings were reported in Germany, where men faced greater risks of severe COVID-19 outcomes and mortality [35]. A higher proportion of observed deaths were registered among divorced individuals in the intrapandemic period. However, without denominator data, this should be interpreted as a descriptive observation rather than an elevated mortality risk.

### Usefulness of the system

While mortality represents a lagging outcome compared with infection or admission data, it can nonetheless generate early epidemiological signals relative to formal diagnostic confirmation, especially in contexts with delayed or incomplete reporting. This conceptualization is consistent with the established use of mortality surveillance as an early warning mechanism in epidemic monitoring [19]. The system’s practical value is demonstrated by the empirical findings: both all-cause and respiratory failure mortality exceeded their 95% prediction intervals during defined pandemic waves. Notably, all-cause mortality showed pronounced peaks in December 2020 and early 2021, while respiratory failure deaths sustained breaches of expected bounds from mid-2020 through 2021. These consistent exceedances—temporally aligned with Egypt’s COVID-19 surges—confirm that even a simplified ETS-based model can capture meaningful outbreak signals. Despite residual autocorrelation indicating imperfect model fit (Ljung–Box *p* = 0.002), the strong temporal correspondence between observed and pandemic peaks underscores the system’s operational sensitivity. Together, the respiratory-specific excess patterns and the broader all-cause mortality deviations illustrate that routine death-registry data can detect both direct and indirect pandemic impacts, supporting their dual role in identifying respiratory-driven outbreaks and monitoring overall health-system stress.

It is important to note that the ≥ 3-month persistence rule was designed as a preliminary screening mechanism rather than a confirmatory outbreak detection algorithm. The intent was to enable field surveillance teams to recognize sustained mortality deviations through simple, reproducible observations. Once flagged, these anomalies can then be subjected to confirmatory analysis or laboratory investigation. Such tiered approaches are well aligned with WHO guidance for stepwise implementation of early warning systems, where initial signals generated from routine data support timely escalation to higher analytic or diagnostic levels [27].

Although April 2020 showed a transient decline—potentially due to reduced fatalities from traffic incidents or other infectious diseases—subsequent sustained increases underscored the system’s capacity for outbreak detection. This aligns with international evidence: in France, Paireau et al. used ensemble models to show excess mortality of 9% in 2020 and 7.5% in 2021 compared to the baseline [36]. Similar trends were reported in the U.S., with COVID-19 driving a 17% increase in overall mortality in 2020 alone [37], and in Italy, which reported nearly 160,000 excess deaths from 2020 to 2021 [38].

Studies in several LMICs have reinforced that simplified mortality surveillance methods—with a focus on real-time, accessible metrics—are both practical and effective. For example, Kenya implemented rapid community-based death reporting and straightforward trend analysis to monitor excess mortality amidst incomplete vital registration and limited analytic capacity [39]. In India, aggregate monthly counts with simple adjustments have been crucial for capturing COVID-19 mortality in settings lacking universal medical certification [40]. Likewise, syndromic surveillance approaches in the Philippines have leveraged basic, rule-based alerts for timely public health responses [41].

In our study, Years of Life Lost (YLL) among adults aged 40–70 due to respiratory failure reached 41,819 over the two pandemic years, with the 65–70 age group contributing the highest burden. The 58% increase in YLL from 2020 to 2021 underscores the growing impact of premature mortality during the pandemic, particularly affecting older working-age adults and young elderly populations.

### Strengths and limitations

This study is the first in Egypt to evaluate a cause-specific mortality surveillance system using official registry data. The integration of both overall and diagnosis-specific mortality, combined with accessible analytic tools, demonstrated how a simplified framework can function as an early warning system in LMIC contexts. The data were obtained from MoHP records and validated against those of the National Central Agency for Public Mobilization and Statistics, supporting the system’s reliability [42].

Our selection of exponential smoothing and a “three-month sustained excess” as surveillance criteria was guided by the constraints of registry infrastructure, digital access, and workforce capacity in Egyptian health offices. While advanced models such as EARS or EuroMOMO offer robust outbreak detection, their implementation typically requires granular data and specialized statistical expertise—resources often unavailable in resource-constrained settings. By prioritizing transparency and rapid applicability, our approach maximizes system adoption and reliability under real-world conditions.

Several limitations should be acknowledged. Despite these strengths, several limitations should be acknowledged. First, while respiratory failure (J96.90) was selected as the main endpoint, broader respiratory categories (J09–J18, J80) were reviewed qualitatively and showed parallel patterns, suggesting that the overall mortality signal was not substantially distorted. The national adoption of U07.x coding occurred late in the pandemic period, as documented in Michael Willie’s 2021 study [23], which justifies their exclusion from the current analysis. However, hospital discharge reports informed death certificate coding, partially mitigating this issue. Diagnostic practices may have shifted during the pandemic (“diagnostic drift”), and reporting delays could have influenced mortality counts. This pragmatic choice reflects the structure of routinely collected registry data available during the study period.

Second, the study was limited to eight urban health offices, precluding analysis of rural-urban disparities in surveillance performance. Third, the poor Ljung–Box fit highlights limitations of formal modeling in this context and reinforces the rationale for adopting simpler, pragmatic thresholds for outbreak detection. Fourth, restricting the analysis of years of life lost (YLL) to ages 40–70 years may underestimate the total YLL burden, particularly among younger populations.

Finally, the proposed methodology is best suited for detecting outbreaks caused by pathogens with predominant respiratory manifestations and may be less sensitive for diseases characterized by non-respiratory mortality patterns. Our approach should be viewed as an initial feasibility assessment designed to inform the operational and methodological requirements for more standardized excess mortality surveillance in similar low-resource settings.

### Research recommendations

To improve the accuracy and timeliness of outbreak detection in low-resource settings, future mortality surveillance systems should prioritize tracking diagnosis-specific deaths, especially those linked to respiratory syndromes, rather than relying solely on all-cause mortality. Building time series from longer historical datasets may enhance the accuracy of baseline mortality trends and improve the detection of significant deviations.

Incorporating objective statistical thresholds—such as z-scores, or cumulative sum (CUSUM) control charts—can support sustained trends by quantifying excess mortality more precisely. Future analyses should employ interrupted time series (ITS) or generalized linear models (GLM) with proper uncertainty intervals for more robust statistical inference, moving beyond simple proportion comparisons. In parallel, investing in basic analytic training for public health personnel will empower local surveillance teams to manage and interpret data independently.

Future studies with access to population-level data should incorporate crude or age-standardized rates to enable fuller comparability with global surveillance systems. This study relied on proportionate mortality rather than crude or age-standardized mortality rates because subdistrict population denominators were not available within the data-sharing scope approved by the Ministry of Health. Notably, even high-income settings have employed proportionate mortality approaches during early system evaluations—for example, in the *Evaluation of the French reactive mortality surveillance system supporting decision making* [43], which also relied on proportional indicators to detect mortality anomalies. Proportionate mortality nonetheless provides meaningful signal detection when applied consistently over time.

Cost-effectiveness analyses are also needed to evaluate the sustainability and impact of mortality surveillance systems in Egypt and other LMICs. These studies should guide strategic investments in digital infrastructure, standardized data protocols, and workforce development, ensuring that surveillance frameworks are both operationally feasible and scalable across diverse healthcare settings. Collectively, these improvements will enhance the responsiveness and resilience of public health systems in the face of emerging infectious disease threats.

## Conclusion

This study suggests that mortality surveillance based on routinely collected cause-of-death data—using proportionate mortality as a feasible first step—can generate useful signals for the detection of respiratory infection outbreaks in LMIC settings. However, operational deployment as an early warning system will require additional work, including standardized thresholds (e.g., EuroMOMO/EARS), routine age-standardized mortality rates, explicit uncertainty measures, and validation against external series such as hospital admissions or national wave patterns. While data quality can be further enhanced, the existing documentation system is sufficient to support early warning functions. Future investments should focus on strengthening human resources, expanding digital infrastructure, and improving data standardization to ensure the long-term sustainability and scalability of mortality surveillance systems in resource-limited settings, especially for respiratory infection outbreaks, where mortality patterns provide timely and actionable public health insights.

## Supplementary Information

Additional file 1. Health office death certificate in Egypt (280 KB).

## Data Availability

The mortality data used in this study originate from official death registry records maintained by the Egyptian Ministry of Health and Population (MoHP). Due to confidentiality and legal constraints governing vital statistics, individual-level data cannot be publicly shared. De‑identified, aggregate monthly counts of deaths by cause, age group, sex, and district (as analyzed in this study) can be made available upon reasonable request to the corresponding author. Access will require obtaining updated ethical approval from the MoHP by the requesting party, followed by a data‑use agreement approved by both the MoHP and the Research Ethics Committee of Alexandria Faculty of Medicine, in line with national data protection and ethics regulations.

## Abbreviations

CDC: Centers for Disease Control and Prevention
COVID-19: Coronavirus Disease 2019
CUSUM: Cumulative Sum Control Chart
EARS: Early Aberration Reporting System
ETS: Error, Trend, Seasonal (exponential smoothing model)
EWS: Early Warning System
GDP: Gross Domestic Product
ICD: International Classification of Diseases
IRB: Institutional Review Board
ITS: Interrupted Time Series
LMICs: Low and Middle Income Countries
LRI: Lower Respiratory Infections
MoHP: Ministry of Health and Population
OPRM: Other Pandemic-Related Mortality
STROBE: Strengthening the Reporting of Observational Studies in Epidemiology
RECORD: The REporting of studies Conducted using Observational Routinely-collected health Data
UNHCR: United Nations High Commissioner for Refugees
WHO: World Health Organization
YLL: Years of Life Lost

## References

[1] Karcıoğlu O, Yüksel A, Baha A, Er AB, Esendağlı D, Gülhan PY, et al. COVID-19: the biggest threat of the 21st century: in respectful memory of the warriors all over the world. Turk Thorac J. 2020;21(6):409–18. 10.5152/TurkThoracJ.2020.20069.​. 10.5152/TurkThoracJ.2020.20069 PMC7752102 33352097

[2] United Nations. Data tells the story on how COVID-19 is changing the world. Department of Economic and Social Affairs. 2024. Available from: https://www.un.org/hi/desa/data-tells-story-how-covid-19-changing-world. Accessed 11 November 2025.

[3] The Economist. The pandemic’s true death toll. The Economist. 2021. Available from: https://www.economist.com/graphic-detail/coronavirus-excess-deaths-estimates. Accessed 2 March 2025.

[4] World Health Organization. 14.9 million excess deaths associated with the COVID-19 pandemic in 2020 and 2021. 2022. Available from: https://www.who.int/news/item/05-05-2022-14.9-million-excess-deaths-were-associated-with-the-covid-19-pandemic-in-2020-and-2021. Accessed 7 February 2025.

[5] Schumacher AE, Kyu HH, Aali A, Abbafati C, Abbas J, Abbasgholizadeh R, et al. Global age-sex-specific mortality, life expectancy, and population estimates in 204 countries and territories and 811 subnational locations, 1950–2021, and the impact of the COVID-19 pandemic: a comprehensive demographic analysis for the global burden of disease study 2021. Lancet. 2024;403(10440):1989–2056. 10.1016/S0140-6736(24)00476-8.​. 10.1016/S0140-6736(24)00476-8 PMC11126395 38484753

[6] United Nations Egypt. Egypt COVID-19 response and recovery interventions of the United Nations in Egypt. 2021. Available from: https://egypt.un.org/en/89430-egypt-covid-19-response-and-recovery-interventions-united-nations-egypt. Accessed 11 November 2025.

[7] Trading Economics. Egypt Coronavirus COVID-19 Deaths. 2023. Available from: https://tradingeconomics.com/egypt/coronavirus-deaths. Accessed 22/4/2025.&#8203.

[8] International Monetary Fund. Egypt: Overcoming the COVID shock and maintaining growth. 2021. Available from: https://www.imf.org/en/News/Articles/2021/07/14/na070621-egypt-overcoming-the-covid-shock-and-maintaining-growth. Accessed 20 April 2025.

[9] Teutsch SM, Thacker SB. Planning a public health surveillance system. Epidemiol Bull. 1995;16(1):1–6. 7794696

[10] Onder G, Rezza G, Brusaferro S. Case-fatality rate and characteristics of patients dying in relation to COVID-19 in Italy. JAMA. 2020;323(18):1775–6. 10.1001/jama.2020.4683. 32203977 10.1001/jama.2020.4683

[11] Dion M, AbdelMalik P, Mawudeku A. Big data and the global public health intelligence network (GPHIN). Can Commun Dis Rep. 2015;41(9):209–14. 10.14745/ccdr.v41i09a02. 29769954 10.14745/ccdr.v41i09a02 PMC5933838

[12] Meckawy R, Stuckler D, Mehta A, Al-Ahdal T, Doebbeling BN. Effectiveness of early warning systems in the detection of infectious diseases outbreaks: a systematic review. BMC Public Health. 2022;22(1):2216. 10.1186/s12889-022-14625-4. 36447171 10.1186/s12889-022-14625-4 PMC9707072

[13] Jayatilleke K. Challenges in implementing surveillance tools of high-income countries in low- and middle-income countries. Curr Treat Options Infect Dis. 2020;12(3):191–201. 10.1007/s40506-020-00229-2. 32874140 10.1007/s40506-020-00229-2 PMC7453076

[14] Wang X, Wu S, MacIntyre CR, Zhang H, Shi W, Peng X, et al. Using an adjusted serfling regression model to improve the early warning at the arrival of peak timing of influenza in Beijing. PLoS ONE. 2015;10(3):e0119923. 10.1371/journal.pone.0119923. 25756205 10.1371/journal.pone.0119923 PMC4354906

[15] Zacher B, Czogiel I. Supervised learning using routine surveillance data improves outbreak detection of Salmonella and Campylobacter infections in Germany. PLoS ONE. 2022;17(5):e0267510. 10.1371/journal.pone.0267510. 35511793 10.1371/journal.pone.0267510 PMC9070876

[16] Badiola-Zabala G, Graña M. On the variability of EuroMOMO reporting of all-cause all-ages mortality z-scores. MedRxiv. 2025;2025–05. 10.1101/2025.05.08.25327238.

[17] Chen H, Zeng D, Yan P. EARS. Infectious disease informatics: syndromic surveillance for public health and biodefense; Editors. New York: Springer; 2009. pp. 167–75.

[18] Abou-Abbas L, Nasser Z, Baaklini M, Cheaito L, Karout J, Sweidan H, et al. COVID-19 mortality surveillance in Lebanon. Sci Rep. 2022;12(1):14639. 10.1038/s41598-022-18715-6. 36030277 10.1038/s41598-022-18715-6 PMC9419139

[19] Rao C, de Savigny D, Atuheire E, Dolan S, Munoz DC, Fat DM, et al. The role of mortality surveillance in pandemic preparedness and response. Bull World Health Organ. 2025;103(3):213–22. 10.2471/BLT.24.292423. 40026663 10.2471/BLT.24.292423 PMC11865855

[20] World Health Organization (WHO). Improving the quality and use of birth, death and cause-of-death information: guidance for a standards-based review of country practices. Geneva: WHO. 2010. Available from: https://www.who.int/publications/i/item/improving-the-quality-and-use-of-birth-death-and-cause-of-death-information. Accessed 7 April 2025.

[21] Lotfy N. Spatial analysis of completeness of death registration in Egypt. J Egypt Public Health Assoc. 2020;95(1):12. 10.1186/s42506-020-00040-3. 32813150 10.1186/s42506-020-00040-3 PMC7364774

[22] Buck CJ. 2016 ICD-10-CM for Physicians Professional Edition. St. Louis (MO): Elsevier Health Sciences. W B Saunders CO; 2015.

[23] Willie MM. Descriptive analysis of factors associated with COVID-19 (U07.1, U07.2), viral pneumonia (J12.8 and J12.9) and other types of admission diagnosis. J Healthc. 2021;4(1):64–9. 10.36959/569/465.

[24] World Health Organization (WHO). WHO methods and data sources for global burden of disease estimates. Geneva: WHO. 2017.​ Available from: https://cdn.who.int/media/docs/default-source/gho-documents/global-health-estimates/ghe2021_daly_methods.pdf . Accessed 3 April 2025.

[25] Balabanova Y, Gilsdorf A, Buda S, Burger R, Eckmanns T, Gärtner B, et al. Communicable diseases prioritized for surveillance and epidemiological research: results of a standardized prioritization procedure in Germany, 2011. PLoS ONE. 2011;6(10):e25691. 10.1371/journal.pone.0025691. 21991334 10.1371/journal.pone.0025691 PMC3186774

[26] Klamer S, Van Goethem N, Working Group D, Criteria Selection G, Muyldermans G, Vernelen K, et al. Prioritisation for future surveillance, prevention and control of 98 communicable diseases in Belgium: a 2018 multi-criteria decision analysis study. BMC Public Health. 2021;21:1–18. 10.1186/s12889-020-09566-9. 33482767 10.1186/s12889-020-09566-9 PMC7820105

[27] Tomov L, Chervenkov L, Miteva DG, Batselova H, Velikova T. Applications of time series analysis in epidemiology: literature review and our experience during COVID-19 pandemic. World J Clin Cases. 2023;11(29):6974–83. 10.12998/wjcc.v11.i29.6974. 37946767 10.12998/wjcc.v11.i29.6974 PMC10631421

[28] Rao C, de Savigny D, Atuheire E, Dolan S, Cobos Munoz D, Fat DM et al. Mortality surveillance for pandemic preparedness and response. Bull World Health Organ. 2025;103(3):213 – 22.​ Available from: https://cdn.who.int/media/docs/default-source/bulletin/online-first/blt.24.292423.pd . Accessed 1 February 2025. 10.2471/BLT.24.292423 PMC11865855 40026663

[29] Mowafi H, Ngaruiya C, O’Reilly G, Kobusingye O, Kapil V, Rubiano AM, et al. Emergency care surveillance and emergency care registries in low-income and middle-income countries: conceptual challenges and future directions for research. BMJ Global Health. 2019;4(Suppl 6):e001442. 10.1136/bmjgh-2019-001442. 31406601 10.1136/bmjgh-2019-001442 PMC6666805

[30] United Nations High Commissioner for Refugees (UNHCR). A Practical Guide for Collecting, Reporting, and Using Surveillance Data for Estimating Mortality in Refugee Settings. Geneva: UNHCR. 2021. Available from: https://www.unhcr.org/sites/default/files/2023-06/guidelines-for-mortality-surveillance.pdf . Accessed 11 November 2025.

[31] Cuschieri S. The STROBE guidelines. Saudi J Anaesth. 2019;13(Suppl 1):S31–4. 10.4103/sja.SJA_543_18. 30930717 10.4103/sja.SJA_543_18 PMC6398292

[32] Benchimol EI, Smeeth L, Guttmann A, Harron K, Moher D, Petersen I, et al. The REporting of studies Conducted using Observational routinely-collected health Data (RECORD) statement. PLoS Med. 2015;12(10):e1001885. 10.1371/journal.pmed.1001885. 26440803 10.1371/journal.pmed.1001885 PMC4595218

[33] Bender RG, Sirota SB, Swetschinski LR, Dominguez R-MV, Novotney A, Wool EE, et al. Global, regional, and National incidence and mortality burden of non-COVID-19 lower respiratory infections and aetiologies, 1990–2021: a systematic analysis from the global burden of disease study 2021. Lancet Infect Dis. 2024;24(9):974–1002. 10.1016/S1473-3099(24)00176-2. 38636536 10.1016/S1473-3099(24)00176-2 PMC11339187

[34] Jiaquan Xu SLM, Kenneth D, Kochanek, Arias E. Mortality in the United States, 2021. National Center for Health Statistics Data Brief, no. 456. Hyattsville (MD): National Center for Health Statistics, Centers for Disease Control and Prevention; 2022. Available from: https://www.cdc.gov/nchs/products/databriefs/db456.htm. Accessed 11 November 2025.

[35] Doerre A, Doblhammer G. The influence of gender on COVID-19 infections and mortality in germany: insights from age- and gender-specific modeling of contact rates, infections, and deaths in the early phase of the pandemic. PLoS ONE. 2022;17(5):e0268119. 10.1371/journal.pone.0268119. 35522614 10.1371/journal.pone.0268119 PMC9075634

[36] Paireau J, Andronico A, Hozé N, Layan M, Crépey P, Roumagnac A, et al. An ensemble model based on early predictors to forecast COVID-19 health care demand in France. Proc Natl Acad Sci USA. 2022;119(18):e2103302119. 10.1073/pnas.2103302119. 35476520 10.1073/pnas.2103302119 PMC9170016

[37] Centers for Disease Control and Prevention (CDC). 2020 final death statistics: COVID-19 as an underlying cause of death vs. contributing cause. National Center for Health Statistics. 2022. Available from: https://www.cdc.gov/nchs/pressroom/podcasts/2022/20220107/20220107.htm. Accessed 27 May 2025.

[38] Alicandro G, Gerli AG, Centanni S, Remuzzi G, La Vecchia C. Excess total mortality in italy: an update to February 2023 with focus on working ages. Med Lav. 2023;114(3):e2023028. 10.23749/mdl.v114i3.14740. 37309878 10.23749/mdl.v114i3.14740 PMC10281071

[39] World Health Organization. Improving Civil Registration, Vital Statistics And Health Data Through Strengthened Partnerships In Kenya., 2022. Available from: https://www.who.int/news-room/feature-stories/detail/strengthening-health-data-kenya. Accessed 11 November 2025.

[40] Shewade HD, Parameswaran GG, Mazumder A, Gupta M. Adjusting reported COVID-19 deaths for the prevailing routine death surveillance in India. Front Public Health. 2021;9:641991. 10.3389/fpubh.2021.641991. 34422738 10.3389/fpubh.2021.641991 PMC8374621

[41] Migriño JR Jr, Batangan ARU, Abello RMR. COVID-19 infection wave mortality from surveillance data in the Philippines using machine learning. MedRxiv. 2023:2023–11. 10.1101/2023.11.28.23299037.

[42] Central Agency for Public Mobilization and Statistics (CAPMAS). Statistical Yearbook. Cairo: CAPMAS. 2023. Available from: https://www.capmas.gov.eg/ . Accessed 11 November 2025.

[43] Baghdadi Y, Gallay A, Caserio-Schönemann C, Fouillet A. Evaluation of the French reactive mortality surveillance system supporting decision making. Eur J Public Health. 2019;29(4):601–7. 10.1093/eurpub/cky251. 30561626 10.1093/eurpub/cky251

