# Supplementary figures and images for: Mortality surveillance as an early warning system for respiratory infection outbreaks: lessons from the COVID-19 pandemic in Alexandria, Egypt
Source: J Egypt Public Health Assoc. 2026 Feb 23;101:4. doi: 10.1186/s42506-025-00205-y (PMC12929754; doi:10.1186/s42506-025-00205-y)

***Health office death certificate in Egypt***


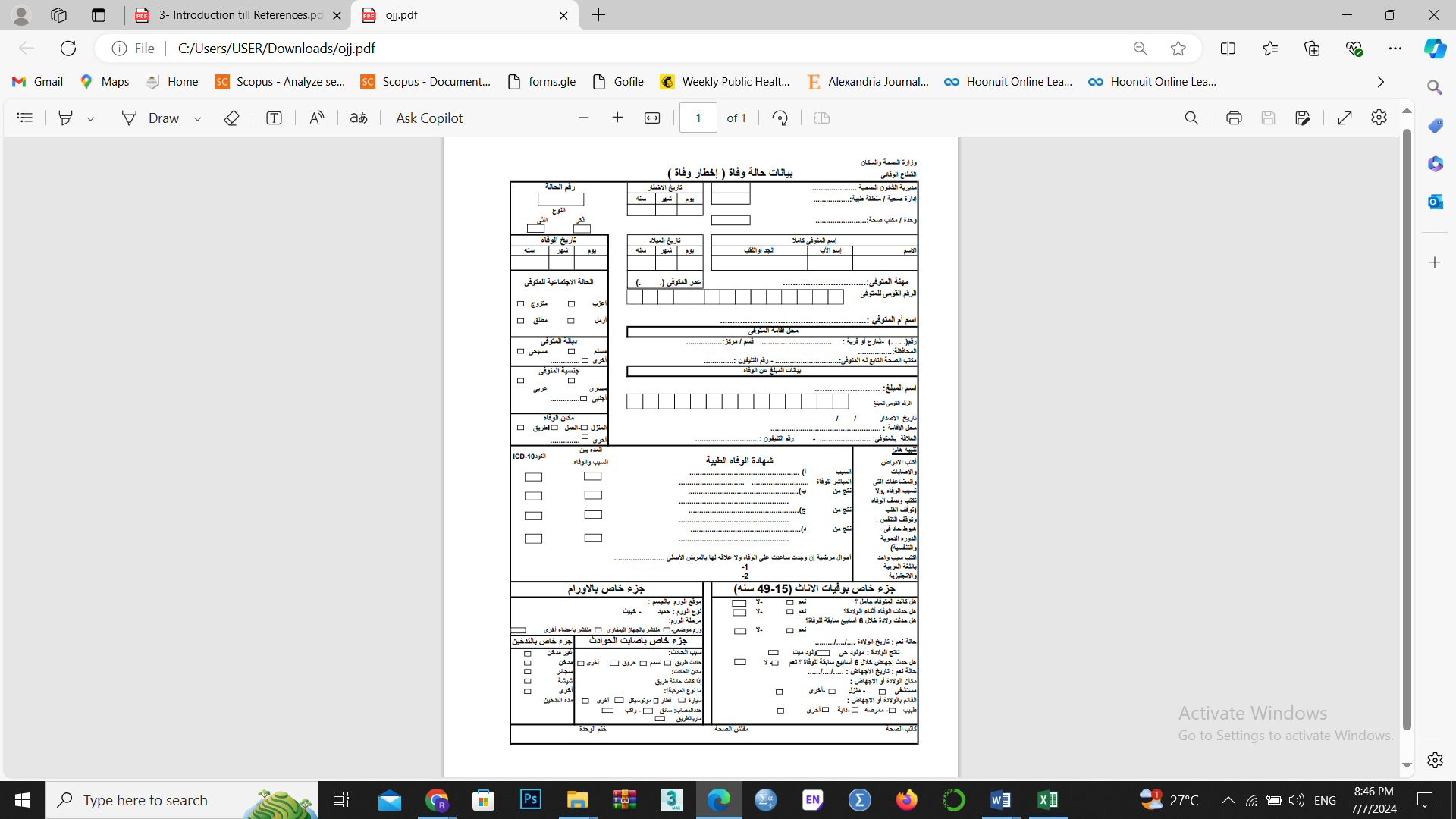

Supplement: Supplementary file 1 — Additional file 1. Health office death certificate in Egypt (280 KB). [file 42506_2025_205_MOESM1_ESM.docx]
